# Pediatric Asthma: A Daily Challenge

**DOI:** 10.3390/children9040576

**Published:** 2022-04-18

**Authors:** Giorgio Ciprandi

**Affiliations:** Allergy Clinic, Casa di Cura Villa Montallegro, 16145 Genoa, Italy; gio.cip@libero.it

Asthma is a prevalent disease in children and adolescents, as the prevalence concerns about 10% of the general pediatric population. Asthma is a chronic disease characterized by airway inflammation, bronchial hyperresponsiveness, and variable airflow obstruction. The management of children and adolescents with asthma is demanding as it should consider multifaceted aspects, including assessment of symptoms, use of medications, measurement of lung function, and inflammation. Asthma treatment should be tailored for every subject. That is, the paradigm is to implement precision medicine to prescribe personalized medicine. In this regard, phenotyping and endotyping patients are mandatory to define the best treatment.

Presently, good control of asthma is the goal of asthma management. Well-controlled asthma requires adequate treatment based on effective and safe medications. Therefore, the measurement of asthma control deserves careful attention in clinical practice and needs appropriate tools. In this regard, the concept of patient-reported outcomes (PRO) acquires a relevant value. Moreover, there is the compelling requirement of adopting pragmatic markers that are simple, economical, reliable, and accessible. In addition, type 2 is the most common phenotype in children and adolescents with asthma. As a result, allergen immunotherapy represents the only causal remedy in allergic subjects.

The present Special Issue includes three articles that explore these issues, with particular relevance to the real-life and clinical practice.

The first paper concerned the measurement of asthma and allergic rhinitis control in children and adolescents [1]. The study’s rationale rests on the widespread association between asthma and allergic rhinitis. Consequently, measuring the control of both diseases requires adequate and validated tools. Combining assessment of both conditions could represent a fruitful edge in daily practice. The Control of Allergic Rhinitis and Asthma Test (CARAT) is a questionnaire that evaluates asthma and allergic rhinitis control. Therefore, the reported study included 138 children and adolescents with asthma and allergic rhinitis recruited at three pediatric allergy centers. Different tools were investigated, including the CARAT, the pediatric version of CARAT (CARATkids), the asthma control test (ACT) and its pediatric version (cACT), and the visual analog scale (VAS) for asthma and nasal symptoms perception.

Moreover, lung function was assessed. Finally, asthma control was assessed using the Global Initiative for Asthma (GINA) criteria. The study showed significant differences between CARAT outcomes and ACT scores in both children and adolescents. These findings were not unexpected as these variables explore different domains of the two diseases. As a result, their use should be complementary. Each test provides information that completes the asthma puzzle.

The second article presented a series of pragmatic biomarkers that may be useful in managing children and adolescents with asthma in daily clinical practice [2]. Many biomarkers have been proposed to define the phenotype and endotype of asthmatic patients. However, most of them only have a research application and are not available for routine use. Peripheral eosinophils and serum allergen-specific IgE are, at present, unique biomarkers available everywhere that provide helpful information. A high eosinophil count suggests the presence of type 2 inflammation. A positive serum test defines a specific sensitization. These two biomarkers allow identifying personalized biological therapy and allergen immunotherapy. In addition, molecular allergy testing provides useful information about genuine allergens and establishes a possible risk of severe allergy.

Furthermore, lung function assessment is mandatory in all subjects with asthma. The demonstration of reversible obstruction, using bronchodilation testing or bronchial challenge, is the main factor leading to asthma diagnosis. In addition, lung function measurement is fundamental to evaluate asthmatic patients over time objectively. In this regard, a forced expiratory flow between 25 and 75% of vital capacity (FEF_25–75_) is a simple parameter that indicates an early bronchial impairment, mainly in subjects with normal FEV_1_ values. That is, it is relatively rare to detect impaired FEV_1_ in young patients, if not in more severe asthma. Finally, nasal cytology may provide interesting information about the type and intensity of airway inflammation as nasal inflammation reflects what happens in the lower airways with good reliability.

The last article considered the pragmatic role of allergen immunotherapy in pediatric asthma [3]. Allergen immunotherapy is the only disease-modifying treatment for allergic asthma. Indeed, children and adolescents experience a significant reduction in symptom severity and medication use after allergen immunotherapy. In addition, allergen immunotherapy significantly improves the quality of life of young patients and their parents. Notably, allergen immunotherapy may prevent asthma onset in subjects with allergic rhinitis. Furthermore, allergen immunotherapy benefits persist after suspension for many years (i.e., carry-over effect). However, allergen immunotherapy requires correct management, including precise diagnosis, choice of a good-quality allergen extract, and thorough follow-up.

In conclusion, the present Special Issue on pediatric asthma offers different perspectives concerning pediatric asthma management in clinical practice. However, there is agreement about the relevant role provided by the asthma control grading. In this regard, it is necessary to recur to validated and plentifully informative instruments, including questionnaires and PRO measures. Moreover, phenotyping and endotyping serve for precise, personalized treatments, but practical biomarkers should be adopted. Finally, allergen immunotherapy represents a disease-modifying therapy of type 2 allergic asthma but requires specialistic knowledge. Therefore, the clinical practice requires validated and suitable tools and remedies.
